# Antigliadin Antibodies (AGA IgG) Are Related to Neurochemistry in Schizophrenia

**DOI:** 10.3389/fpsyt.2017.00104

**Published:** 2017-06-19

**Authors:** Laura M. Rowland, Haley K. Demyanovich, S. Andrea Wijtenburg, William W. Eaton, Katrina Rodriguez, Frank Gaston, Daniela Cihakova, Monica V. Talor, Fang Liu, Robert R. McMahon, L. Elliot Hong, Deanna L. Kelly

**Affiliations:** ^1^Maryland Psychiatric Research Center, Department of Psychiatry, University of Maryland School of Medicine, Baltimore, MD, United States; ^2^Department of Mental Health, Johns Hopkins Bloomberg School of Public Health, Baltimore, MD, United States; ^3^Immunologic Disorders Laboratory, Department of Pathology, Johns Hopkins University School of Medicine, Baltimore, MD, United States

**Keywords:** gluten, gliadin, antibody, myoinositol, GPC + PC, schizophrenia, neuroimaging, inflammation

## Abstract

Inflammation may play a role in schizophrenia; however, subgroups with immune regulation dysfunction may serve as distinct illness phenotypes with potential different treatment and prevention strategies. Emerging data show that about 30% of people with schizophrenia have elevated antigliadin antibodies of the IgG type, representing a possible subgroup of schizophrenia patients with immune involvement. Also, recent data have shown a high correlation of IgG-mediated antibodies between the periphery and cerebral spinal fluid in schizophrenia but not healthy controls, particularly AGA IgG suggesting that these antibodies may be crossing the blood–brain barrier with resulting neuroinflammation. Proton magnetic resonance spectroscopy (MRS) is a non-invasive technique that allows the quantification of certain neurochemicals *in vivo* that may proxy inflammation in the brain such as myoinositol and choline-containing compounds (glycerophosphorylcholine and phosphorylcholine). The objective of this exploratory study was to examine the relationship between serum AGA IgG levels and MRS neurochemical levels. We hypothesized that higher AGA IgG levels would be associated with higher levels of myoinositol and choline-containing compounds (glycerophosphorylcholine plus phosphorylcholine; GPC + PC) in the anterior cingulate cortex. Thirty-three participants with a DSM-IV diagnosis of schizophrenia or schizoaffective disorder had blood drawn and underwent neuroimaging using MRS within 9 months. We found that 10/33 (30%) had positive AGA IgG (≥20 U) similar to previous findings. While there were no significant differences in myoinositol and GPC + PC levels between patients with and without AGA IgG positivity, there were significant relationships between both myoinositol (*r* = 0.475, *p* = 0.007) and GPC + PC (*r* = 0.36, *p* = 0.045) with AGA IgG levels. This study shows a possible connection of AGA IgG antibodies to putative brain inflammation as measured by MRS in schizophrenia.

## Introduction

Several emerging lines of evidence suggest that the etiology and pathophysiology of schizophrenia may be related to inflammatory processes. Data contributing to this hypothesis include prenatal maternal infection and the subsequent pro-inflammatory response (1–3). Also, multiple studies have demonstrated increased levels of various peripheral cytokines to be elevated in people with first-episode or multi-episode schizophrenia (1, 2, 4–8). In addition, positron emission tomography (PET) studies have demonstrated increased binding to the 18-kDa translocator-protein (TPSO; a marker of microglial activation) in the brains of people with schizophrenia (9–11). Finally, several genome-wide association studies have documented the presence of single-nucleotide polymorphisms in the major histocompatibility complex, genes related to immune function, that are associated with increased risk of schizophrenia (12–17).

A subset of individuals with schizophrenia may be particularly sensitive to inflammation due to immune activation to specific antigens, and this may contribute to the illness pathophysiology. This is in line with the fact that studies on inflammatory markers are not elevated in all people with schizophrenia and why inconsistent results have been shown in cross-sectional cytokine studies. The exacerbation of systemic or brain immune activation could be due to increased permeability of the mucosal epithelial tight junctions in the intestine and blood–brain barrier (18–21). Increased permeability permits entrance of pathogens, toxins, and antigens that could lead to subsequent immune response and reaction; a postulated mechanism of the brain to gut relationship mediated by inflammation. Partial support comes from a recent study indicating increased blood–cerebral spinal fluid (CSF) permeability coupled with antibody response to dietary proteins in first-episode schizophrenia (22). This study found a high correlation of IgG-mediated antibodies (e.g., antibodies to gliadin) between the periphery and CSF in schizophrenia but not healthy controls.

Positivity to immunoglobulin G antibodies to gliadin (AGA IgG) are observed in about 20–30% of people with schizophrenia compared to less than 10% in healthy controls (23–25). This potentially reflects gluten sensitivity (GS), which is a newly characterized syndrome defined by some intestinal but mostly extra-intestinal symptoms related to the ingestion of gluten-containing food (i.e., wheat, barley, or rye) distinct from celiac disease (CD) and wheat allergy (26, 27). High levels of AGA IgG have also been observed in brain conditions such as ataxia (28–30). This provides further support for the gut–brain inflammation linkage. It is plausible that there is a subset of about one-quarter to one-third of the schizophrenia population that may be highly susceptible to GS-mediated peripheral and central inflammation.

Proton magnetic resonance spectroscopy (MRS) is a non-invasive technique that allows the quantification of certain neurochemicals *in vivo*. These neurochemicals reflect a wide variety of mechanisms that range from neuronal function to neurotransmission. MRS biochemicals such as myoinositol and glycerophosphorylcholine (GPC) plus phosphorylcholine (PC) referred to as “GPC + PC” may serve as a proxy for inflammation. Myoinositol is localized primarily in glial cells (31) and is elevated in conditions characterized by central nervous system inflammation (32) such as hepatitis C virus-associated encephalopathy in occipital and parietal gray and white matter (33) and multiple sclerosis in white and cortical gray matter (34, 35). GPC + PC levels putatively reflect cellular membrane metabolism, both synthesis and breakdown (36, 37), and increased levels are observed in neuroinflammatory diseases (32). Hence, spectroscopic measures of myoinositol and GPC + PC could provide insight into brain inflammation. With respect to schizophrenia, results are inconsistent regarding alterations in myoinositol and GPC + PC levels in various brain regions (38, 39). However, the majority of studies on schizophrenia did not examine the immune or measures related to inflammation except for one study that reported higher medial temporal lobe myoinositol in a subset of patients with elevated S100B (40).

The objective of this exploratory study was to examine the relationship between serum AGA IgG levels and MRS neurochemical measurements of the anterior cingulate cortex (ACC) in schizophrenia. Given that AGA IgG antibodies may cross the blood–brain barrier in schizophrenia (22), we hypothesized that those who tested positive for AGA IgG would have higher levels of ACC myoinositol and GPC + PC compared to those who tested negative for AGA IgG. Furthermore, we hypothesized that higher AGA IgG levels would be associated with higher levels of ACC myoinositol and GPC + PC. The ACC was the focus since it is involved in the pathophysiology of schizophrenia, as strongly supported by MRS, other neuroimaging modalities, and postmortem research (41, 42).

## Materials and Methods

Participants were recruited from the Maryland Psychiatric Research Center. Three hundred sixty-six patients with a DSM-IV-TR diagnosis of schizophrenia or schizoaffective disorder participated in a study to measure serum AGA IgG. Of those who completed the antibody screening, 33 participants were able to also complete an MRS session; inclusion criteria for the MRS portion consisted of those between the ages of 18 and 55, and without contraindications for MR scanning (e.g., claustrophobia, metal contained in their bodies). Participants had the MRS scan and blood draw on separate occasions averaging approximately 8.8 months apart and no patient was on a gluten-free diet. All participants were evaluated for their capacity to provide informed consent before giving written consent prior to participation. The University of Maryland Baltimore Institutional Review Board approved this study.

### Anti-Gliadin Antibodies

Blood was drawn and serum stored at −80°F for batched analysis at the Johns Hopkins University Immune Disorders Laboratory. The sera were analyzed for AGA IgG using the INOVA kit #708650, which is an ELISA measure for native gliadin, not a deaminated version of gliadin (linked to CD). We utilized positivity of AGA IgG according to the manufacturer cutoff of AGA IgG levels ≥20 U. We have also independently replicated the cutoff value in a sample of over 370 people with schizophrenia compared to 80 healthy controls with no psychiatric or medical comorbidities. Ninety percent of healthy controls fall below the cutoff and the distribution in schizophrenia is bimodal showing means in those not positive to be <10 U and the mean values of those who are positive to be >40 U (Cihakova et al., under review).

### MRS Acquisition and Analysis

**Figure 1:**
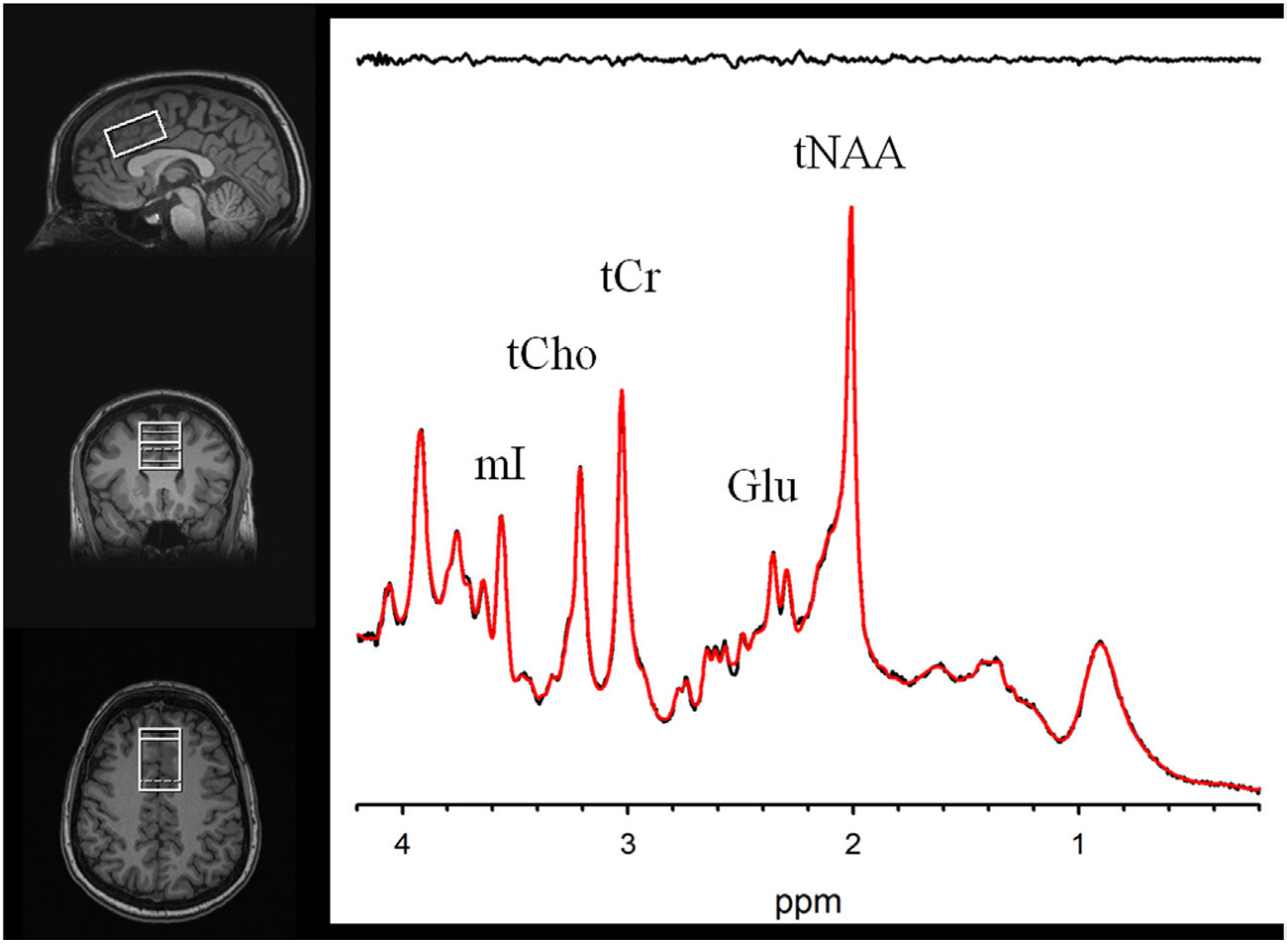
Voxel images and a representative spectrum (black line), LCModel fit (red line), residual (black line at top). Myoinositol (mI), Phosphocreatine+Creatine (tCr), Phosphorylcholine+Glycerophosphorylcholine (tCho), Glutamate (Glu), and N-Acetylaspartate (tNAA).

MR scanning was conducted on a 3-T Siemens Tim Trio equipped with a 32-channel head coil. Head position was fixed with foam padding to minimize movement. Anatomical T1-weighted images were acquired for spectroscopic voxel placement with a “MP-RAGE” sequence. Spectroscopic methods have been previously described (43). The spectroscopic voxel was 4.0 cm × 3.0 cm × 2.0 cm prescribed on the midsagittal slice and positioned parallel to the genu of the corpus callosum and scalp with the midline of the voxel corresponding to the middle of the genu of the corpus callosum. The voxel contained a mixture or rostral and dorsal ACC. Spectra were acquired with a phase rotation STEAM: TR/TM/TE = 2,000/10/6.5-ms, VOI ~24 cm^3^, NEX = 128, 2.5-kHz spectral width, 2,048 complex points, and phases: φ1 = 135°, φ2 = 22.5°, φ13 = 112.5°, φADC = 0°. The test–retest reproducibility of this sequence is excellent, as reported in both healthy volunteers (44) and participants with schizophrenia (45). A water reference (NEX = 16) was also acquired for phase and eddy current correction as well as quantification. LCModel (6.3-0D) was used for spectral quantification (46) with a simulated basis set that contained alanine (Ala), aspartate (Asp), creatine (Cr), γ-aminobuytric acid (GABA), glucose (Glc), glutamate (Glu), glutamine (Gln), glutathione (GSH), glycine (Gly), glycerophosphocholine (GPC), lactate (Lac), myo-Inositol (mI), *N*-acetylaspartate (NAA), *N*-acetylaspartylglutamate (NAAG), phosphocholine (PC), phosphocreatine (PCr), phosphoroylethanolamine (PE), scyllo-Inositol (sI), and taurine (Tau). Metabolite levels are reported in institutional units, and metabolites with Cramer Rao Lower Bounds ≤20% were included in further analyses. The spectroscopic voxel was segmented into gray, white, and CSF tissues using SPM8 and in house MATLAB code, and the metabolite levels were corrected for the proportion of gray, white, and CSF tissue proportions (44). The following metabolites were quantified and reported: total choline (glycerophosphorylcholine + phosphorylcholine), myoinositol, glutamine, glutamine, glutamate + glutamine, glutathione, *N*-acetylaspartate, and total creatine (creatine + phosphocreatine). Spectroscopic voxel location and corresponding spectrum are illustrated in Figure 1.

### Statistical Analyses

Data were not normally distributed and therefore non-parametric statistical analyses were conducted. The differences in MRS measures between those patients who were “positive” (i.e., >20 U) vs. “negative” for the AGA IgG antibodies were examined with the Kruskal–Wallis test. Spearman’s correlations were performed between the MRS measures and AGA IgG levels on all participants.

## Results

A description of the clinical and demographic information is listed in Table 1. Briefly, the mean age of the participants was 33.8.36 ± 12.4 (SD), 52% were African-American, and 48% were Caucasian. Of these, there were 18 were males (55%) and 15 females (45%). Participants were of mixed illness duration with the majority (*N* = 28) having been ill for more than 2 years. Ten of the 33 participants were positive for AGA IgG GS (30%). There were no significant differences in demographic information of those with and without positivity to IgG AGA. The overall mean IgG AGA level in the group was 17.03 ± 24.29 U. The mean AGA IgG in the positive group was 44.61 ± 29.09 vs. 5.04 ± 4.02 U in the AGA IgG negative group (*t* = 20.30, df = 1, *p* < 0.0001).

**Table 1:** Demographic and clinical information.

Variable	Overall group (*N* = 33)	AGA IG positive group (*N* = 10)	AGA IgG negative group (*N* = 23)	Statistic between groups
Mean age (years)	33.8 ± 12.4	32.0.1 ± 11.3	34.6 ± 13.1	*T* = 0.30, *p* = 0.58
Sex (male)	18 (55%)	6 (60%)	12 (52%)	χ^2^ = 0.17, *p* = 0.68
Race				
African-American	17 (52%)	6 (60%)	11 (48%)	χ^2^ = 0.41, *p* = 0.52
Caucasian	16 (48%)	4 (40%)	12 (52%)	
Schizophrenia vs. schizoaffective diagnosis	25 (76%) 8 (24%)	7 (70%) 3 (30%)	18 (78%) 5 (22%)	χ^2^ = 0.26, *p* = 0.61	
Duration of illness (years)	16.2 ± 14.8 (*N* = 31)	12.4 ± 12.1 (*N* = 9)	16.2 ± 14.8 (*N* = 22)	*T* = 0.7, *p* = 0.5
Mean AGA IgG (U)	17.0 ± 24.3	44.6 ± 29.1	5.0 ± 4.0	*T* = 20.3, *p* < 0.0001

**Figure 2:**
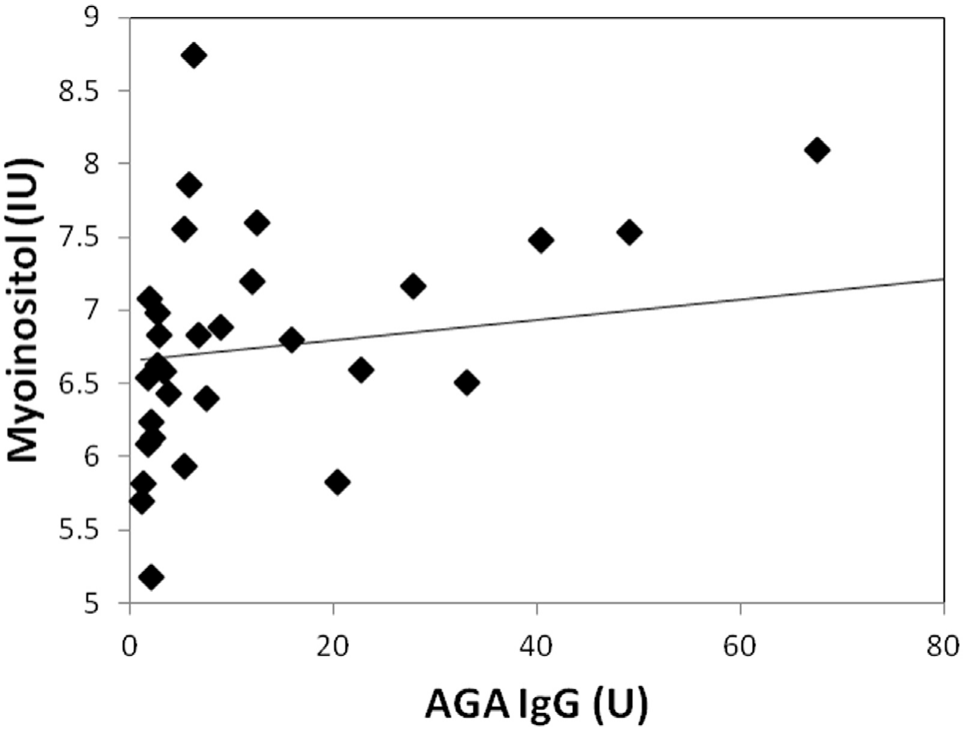
Correlation plot showing a strong positive relationship (*r* = 0.48, *p* = 0.007) between AGA IgG and myoinositol levels. 31/33 of the participants had data available.

**Figure 3:**
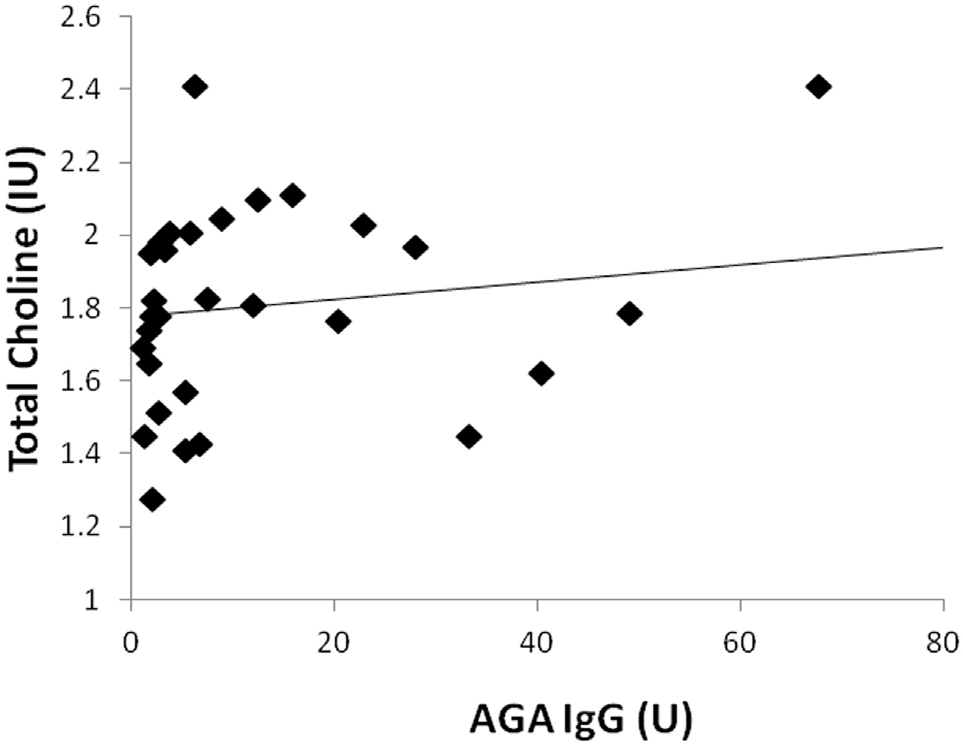
Correlation plot showing a positive relationship (*r* = 0.36, *p* = 0.045) between AGA IgG and GPC + PC levels. 31/33 of the participants had data available.

Two participants moved during MR scanning and therefore spectral quality was poor and not included in the analysis. Contrary to hypothesis, there were no significant differences in myoinositol or GPC + PC between participants who were AGA IgG positive compared to those who were IgG negative. There were no differences in the other metabolites between AGA IgG-positive and -negative groups. The correlation analyses between AGA IgG and the metabolites are reported in Table 2. Results revealed significant relationships between AGA IgG levels and both myoinositol (*r* = 0.475, *p* = 0.007) and GPC + PC (*r* = 0.36, *p* = 0.045) (see Figures 2 and 3). There were no significant correlations noted for glutamate, glutamine, glutathione, *N*-acetylaspartate, or creatine (*p* > 0.1, Table 2).

**Table 2:** Spearman’s correlations between magnetic resonance spectroscopy measures and AGA IgG in schizophrenia patients.

Metabolite	Mean (IU)	SD	*r*-Value	*p*-Value
Glutamate (*N* = 31)	9.12	0.87	0.104	0.58
Glutamine (*N* = 30)	2.18	0.39	−0.1048	0.58
Glutamate + glutamine (*N* = 31)	10.97	1.21	0.006	0.97
Glutathione (*N* = 31)	2.11	0.35	0.01	0.96
*N*-acetylaspartate (*N* = 31)	9.91	0.90	0.006	0.97
Total creatine (*N* = 31)	8.87	0.92	0.25	0.18
Myoinositol (*N* = 31)	6.76	0.77	0.48	0.007
Glycerophosphorylcholine + phosphorylcholine (*N* = 31)	1.81	0.27	0.36	0.045

## Discussion

Despite the accumulating evidence of the evidence of AGA IgG antibodies in a subset of schizophrenia, little work has shown the connection of these antibodies with brain inflammation. To the best of our knowledge, this is the first study to show a relationship between peripheral AGA IgG and ACC myoinositol and GPC + PC levels. Our results also replicate the estimated prevalence of AGA IgG positivity in approximately one-third of people with schizophrenia.

Magnetic resonance spectroscopy measures of brain myoinositol and GPC + PC are thought to reflect inflammation in the brain. Myoinositol is a glial cell marker (31) and is elevated in conditions characterized by CNS inflammation such as HCV-associated encephalopathy, HIV, and multiple sclerosis (33–35, 47). Choline-containing compounds such as GPC + PC reflect cellular membrane synthesis and breakdown (37), and high levels of GPC + PC have been observed in multiple sclerosis with active lesions (48, 49) and HIV infection (50). One study reported higher MRS myoinositol and GPC + PC levels coupled with higher microglial activation measured with PET in patients with hepatitis C (51). The combination of these studies provides good support that MRS measures of myoinositol and GPC + PC proxy neuroinflammation (32). Therefore, the association between higher levels of AGA IgG and brain myoinositol and GPC + PC suggests a link between AGA IgG and CNS inflammation in schizophrenia. It is important to note that the relationships between AGA IgG and the brain metabolites were specific to myoinositol and GPC + PC only, further supporting the AGA IgG neuroinflammation link. These results are also consistent with a study showing a high-positive correlation between blood AGA IgG and CSF AGA IgG in schizophrenia patients but not healthy controls (22), suggesting greater CNS permeability and likely inflammation.

The ACC was the focus of this study because of its involvement in the pathophysiology of schizophrenia supported by postmortem and imaging research (41, 42). Volumetric MRI studies suggest that both dorsal and rostral ACC gray matter is reduced in schizophrenia (41), and proton MRS studies suggest ACC glutamatergic and GABAergic alterations in schizophrenia (42, 52, 53). Postmortem work parallels these imaging findings as indicated by reduced ACC neuropil and altered GABAergic and glutamatergic neurons (41) in schizophrenia. If inflammation is a contributing factor to these ACC alterations is not clear, as studies focused on inflammatory postmortem and PET markers in cortical regions including the ACC have been inconsistent (54). Moreover, the majority of MRS studies of the ACC in schizophrenia did not report alterations in myoinositol or GPC + PC (38); however, previous studies also did not examine specific immune parameters. It is also unclear how the current study’s findings translate to other brain regions. Future studies are necessary to determine if AGA IgG is related to MRS myoinositol and GPC + PC in other brain regions.

Several study limitations are worth mentioning. First, the blood draw for AGA IgG was not on the same day as the imaging procedures; however, due to a long half-life of serum IgG antibodies (~20 days) (55) and long-term stability of AGA IgG in patients with schizophrenia (<15% change in 6 months) (Kelly, unpublished data), we do not anticipate significant changes of AGA IgG levels. Second, as with the majority of studies in schizophrenia, all patients were taking antipsychotic medications, which could impact the results. We do not have the specific antipsychotic medication to include in the report. However, data support that immune involvement is independent of antipsychotic treatment (56–66). Additionally, antipsychotic medication treatment in rodents does not change GPC + PC or myoinositol levels (67). Third, we do not have clinical symptom status at the time of blood draw to link to symptomatology and the groups were too small to do subanalysis by gender, age, or illness duration. Only one brain region was examined, therefore brain region specificity cannot be discussed. Fourth, since causation cannot be determined from this data, it remains unknown if this sensitivity to gluten causes neuroinflammation or if antibodies are high based on neuroinflammatory process present in this group. Preclinical research is needed to determine potential causative factors related to these linked phenomena. Finally, it is not possible to ascertain if higher GPC + PC reflects cell membrane formation or breakdown, so the results should be interpreted with caution.

In conclusion, these results suggest a possible connection of AGA IgG antibodies to putative brain inflammation as measured by MRS in schizophrenia. More research is needed to help delineate the group of people at risk for GS, which is likely a subset of schizophrenia. It is possible that interventions targeted to reduce the immune response to gluten, such as a gluten-free diet, could prove beneficial to ameliorating neuroinflammation and possibly illness symptoms.

## Ethics Statement

All participants were evaluated for their capacity to provide informed consent before giving written consent prior to participation. This study was approved by the University of Maryland Baltimore Institutional Review Board.

## Author Contributions

LR, DK, SW, WE, KR, and DC conceptualized and designed the project. HD, SW, MT, DK, and LH acquired the data. FG, FL, SW, LR, RM, DC, KR, and DK analyzed the data. LR, HD, SW, WE, KR, FG, DC, MT, FL, RM, LH, and DK interpreted the study results, drafted the manuscript and revised it critically, and gave final approval.

## Conflict of Interest Statement

The authors declare that the research was conducted in the absence of any commercial or financial relationships that could be construed as a potential conflict of interest.
